# Universal relation for life-span energy consumption in living organisms: Insights for the origin of aging

**DOI:** 10.1038/s41598-022-06390-6

**Published:** 2022-02-21

**Authors:** Andrés Escala

**Affiliations:** Departamento de Astronomía, Universidad de Chile, Casilla 36-D, Santiago, Chile

**Keywords:** Biophysics, Ecology

## Abstract

Metabolic energy consumption has long been thought to play a major role in the aging process (Pearl, The rate of living. University of London Press, London, 1928). Across species, a gram of tissue expends approximately the same amount of energy during the lifespan on average (Speakman, J Exp Biol 208:1717–1730, 2005). Energy restriction has also been shown to increase the maximum lifespan (McCay et al. J Nutr 10:63–79, 1935) and to retard age-associated changes (Weindruch and Walford, The retardation of aging and disease by dietary restriction. CC Thomas, Springfield, 1988). However, there are significant exceptions to universal energy consumption during the lifespan, mainly found by interclass comparison (Ramsey et al. Free Rad Biol Med 29:946–968, 2000; Atanasov, Trakia J Sci 10(3):1–14, 2012). Here, we present a universal relation that relates lifespan energy consumption to several physiological variables, such as body mass, temperature and the ratio of heart rate to respiratory rate, which have been shown to be valid for $\sim 300$ species representing different classes of living organisms, from unicellular organisms to the largest mammals. This relation has an average scattered pattern restricted to factors of 2, with 95% ($2-\sigma$) of the organisms having departures of less than a factor of $\pi$ from the relation, despite the difference of $\sim 20$ orders of magnitude in body mass, reducing any possible interclass variation in the relation to only a geometrical factor. This result can be interpreted as supporting evidence for the existence of an approximately constant total number ${\mathrm{N}}_{{\mathrm{r}}} \sim 10^8$ of respiration cycles per lifetime for all organisms studied, effectively predetermining the extension of life through the basic energetics of respiration (quantified by ${\mathrm{t}}_{{\mathrm{life}}} = \mathrm{N}_\mathrm{r}/\mathrm{f}_{{\mathrm{resp}}}$); this is an incentive to conduct future studies on the relation of such a constant number ${\mathrm{N}}_{\mathrm{r}}$ of cycles per lifetime due to the production rates of free radicals and oxidants or alternative mechanisms, which may yield definite constraints on the origin of aging.

## Introduction

Aging seems to be an inherent characteristic of all living organisms; while some inert objects can easily subsist on Earth for many centuries, living systems have a much narrower existence, typically limited to decades for large animals (with few exceptions such as Hydra). It is then somewhat natural to try to associate the process of aging with metabolism, since all living organisms obtain the energy required to stay alive from such a process. Rubner, in 1908^7^, compared the energy metabolisms and lifespans of five domestic animals (guinea pig, cat, dog, cow and horse) and humans and found that the lifespan (total) energy expenditure per gram for the five species is approximately constant, suggesting that the total metabolic energy consumption per lifespan is fixed, which later became known as the ‘rate of living’ theory^1^.

Decades later, a mechanism was found by which the idea behind a fixed energy consumption per lifespan might operate, the ‘free-radical damage’ hypothesis of aging^8,9^, in which the macromolecular components of the cell are under perpetual attack from toxic byproducts of metabolism, such as free radicals and oxidants. However, the ‘free-radical’ theory has lost support in recent years^36^, with evidence that a reduction in free radicals by antioxidant supplementation in the diets of laboratory animals does not significantly increase their life expectancy. On the other hand, caloric (energy) restriction has been experimentally shown to increase the maximum lifespan and retard age-associated changes in animals, such as insects, rats, fish, spiders, water fleas and mice^3,4^, as expected by the ‘rate of living’ theory, but it has also been shown to have varying effects on both the metabolic rate and lifespan of other species^20^.

Rubner’s relation was partially confirmed for approximately one hundred mammals^10^ and was extended to birds^11^, ectotherms^12^ and even unicellular organisms such as protozoa and bacteria^6^, totaling almost three hundred different species in a range of 20 orders of magnitude in body mass. Although the total metabolic energy exhausted per lifespan per body mass of a given species appears to be a relatively constant parameter—approximately the same number determined by Rubner^7^, a million Joules per gram of body weight for mammals—variations of more than an order of magnitude have been found among different animal classes; this result has also been found by other authors (i.e.,^2^) and is considered the most persuasive evidence against the ‘rate of living’ theory^5^. Moreover^25^, recently studied Rubner’s relation for a much larger dataset that includes all eukaryotes, finding again that the total metabolic energy exhausted per lifespan per body mass is no longer approximately constant among different animal classes and instead increases allometrically with body mass. A summary of the evidence contradicting the rate of living theory can be found in^26^.

The origin of such variations in the lifespan energy consumption might come from intrinsic variations in the quantities used to estimate it: lifespans and in particular the metabolic rate relation, the universality of which is still debated (see^13^ or^26^ and the references therein). Recently, the empirical metabolic rate relation was corrected to satisfy dimensional homogeneity^13^, a minimal requirement of any meaningful law of nature^14^, and a new metabolic rate (B) formula was proposed: ${\mathrm{B}} = \epsilon ({\mathrm{T}}) \, \eta _{{\mathrm{O}}_2} {\mathrm{f}}_{\mathrm{H}} \, {\mathrm{M}}$, where M is the body mass, ${\mathrm{f}}_{\mathrm{H}}$ is a characteristic (heart) frequency, $\eta _{{\mathrm{O}}_2}$ is a specific $\mathrm {O_2}$ absorption factor and $\epsilon ({\mathrm{T}})=\epsilon _0 \, {{\mathrm{e}}^{\small -{\mathrm{E}}_{{\mathrm{a}}}/{\mathrm{k}} {\mathrm{T}}}}$ is a temperature correction inspired by the Arrhenius formula, in which ${\mathrm{E}}_{\mathrm{a}}$ is the activation energy and k is the Boltzmann universal constant. Compared to Kleiber’s original formulation^15^, ${\mathrm{B}} = {\mathrm{B}}_0 ({\mathrm{M/M}}_0)^{0.75}$, this new metabolic rate relation takes the heart frequency as a controlling variable (i.e., ${\mathrm{f}}_{\mathrm{H}}$ is a marker of the metabolic rate), and it has the advantage of being a unique metabolic rate equation for different classes of animals and different exercise conditions that is valid for both basal and maximal metabolic rates, in agreement with the empirical data in the literature^13^.

In addition, this new metabolic rate relation can be directly linked to the total energy consumed in a lifespan^13^, which is a promising way to explain the origin of the variations in Rubner’s relation and unify them into a single formulation. In this paper, we will explore the implications of this new metabolic relation for the total energy consumed in a lifespan and the ‘rate of living’ theory. The paper is organized as follows: first, we review the results of the new metabolic rate relation found in^13^ and derive its prediction for the total energy consumed in a lifespan in "New metabolic rate relation and its predictions for the ‘rate of living’ theory"; second, in "Empirical support for the corrected relation of the total energy consumed in a lifespan", we continue by testing the empirical support of the predicted relation for total energy consumed in a lifespan, with satisfactory results; and finally, in "Conclusions and outlook", we discuss the results and implications of this work.

## New metabolic rate relation and its predictions for the ‘rate of living’ theory

In the proper mathematical formulation of natural laws, a *minimum* requirement is that the law be expressed in a general form that remains true when the size of units is changed; this is because nature cannot fundamentally depend on a human construct such as the definition of units. In mathematical terms, this implies that meaningful laws of nature must be homogeneous equations in various units of measurement^14^. However, most relations in allometry do not satisfy this basic requirement, including Kleiber’s ‘3/4 Law’^15^, which relates the basal metabolic rate and body mass as ${\mathrm{B}} = {\mathrm{B}}_0 \, ({\mathrm{M/M}}_0)^{3/4} = \mathrm{C} \, {\mathrm{M}}^{3/4}$; this ‘3/4 Law’ is a typical example^14^ of a relation in which the proportionality factor C has a fractal dimensionality, and its value depends on the units chosen for the variables (${\mathrm{B, M}}$), so it does not fulfill the *minimum* requirement for being a natural law. Mathematically speaking, to qualify as a natural law, the metabolic rate relation must first be rewritten so that the constants with dimensions are universal and are restricted to a minimum number, which in no case can exceed the total number of fundamental units of the problem.

To address this issue, we propose a new unique homogeneous equation for metabolic rates that includes the heart frequency ($\mathrm{f}_{\mathrm{H}}$) as a controlling variable^13^; the proposed metabolic rate (B) formula is ${\mathrm{B}} = \epsilon ({\mathrm{T}}) \, \eta _{{\mathrm{O}}_2} \mathrm{f}_{\mathrm{H}} \, {\mathrm{M}}$, where M is the body mass, $\mathrm{f}_{\mathrm{H}}$ is a characteristic (heart) frequency, $\eta _{{\mathrm{O}}_2}$ is a specific absorption factor ${{\mathrm{O}}_2}$ and $\epsilon ({\mathrm{T}})$ is temperature-dependent normalization. The new metabolic rate relation is, in addition to being in agreement with the empirical data^13^, valid for different classes of animals and for both resting and exercising conditions. Using this formula, it can be shown that most of the differences found in the allometric exponents are due to comparing incommensurable quantities^13^; the variations in the dependence of the metabolic rates on body mass are secondary, as they come from variations in the allometric dependence of heart frequencies on body mass. Therefore, $\mathrm{f}_{\mathrm{H}}$ can be mathematically seen as a new physiological variable that controls metabolic rates, in addition to body mass, only in the sense that metabolic rates and heart frequencies must covary for physiological reasons: an increase (or decrease) in metabolism (i.e., due to starting or stopping exercise) must be accompanied by an increase (or decrease) in heart frequency after a latency period to support long-term increased (or decreased) activity.

One of the advantages of having a metabolic rate relation with ${\mathrm{f}}_{{\mathrm{H}}}$ as a controlling physiological variable is that it can be straightforwardly linked^13^ to the total energy consumed in a lifespan through the relation of the total number ${\mathrm{N}}_{\mathrm{b}}$ of heartbeats in a lifetime, ${\mathrm{N}}_{\mathrm{b}} = \mathrm{f}_{{\mathrm{H}}} \, \mathrm{t}_{{{\mathrm{life}}}}$, which is empirically determined to be relatively constant across mammalian species and equals $7.3 \times 10^8$ heartbeats^16^. Therefore, for a constant total number of heartbeats in a lifetime, ${\mathrm{t}}_{{{\mathrm{life}}}} = \mathrm{N}_{\mathrm{b}} \, / \, \mathrm{f}_{{\mathrm{H}}}$, the metabolic rate relation^13^ can be rewritten as ${\mathrm{B}} \, \mathrm{t}_{{\mathrm{life}}} = \epsilon (\mathrm{T}) \, \eta _{{{\mathrm{O}}}_2} \mathrm{N}_{{\mathrm{b}}} \, {\mathrm{M}}$, giving a straightforward prediction of lifespan energy consumption that can be seen as a test of the ‘rate of living’ theory^1^.

However, since the relation of the total energy consumed in a lifespan exists for all eukaryotes^25^, even including unicellular organisms without hearts^6^, to find a unique relation for all living organisms, the concept of the characteristic frequency must first be generalized. A natural candidate is the respiration frequency, ${\mathrm{f}}_{{\mathrm{resp}}}$, since this frequency is observed in animals to be strictly proportional to the heart frequency, $\mathrm{f}_{\mathrm{H}} = \mathrm{a} \, \mathrm{f}_{{{\mathrm{resp}}}}$^17^, and is still a meaningful frequency for organisms without hearts. Under this proportionality between frequencies, the empirical relation for a lifetime can also be rewritten to be valid for a total number $\mathrm{N}_{{\mathrm{r}}} \,(=\mathrm{N}_{{\mathrm{b}}}/{{\mathrm{a}}})$ of ‘respiration cycles’: $\mathrm{t}_{{\mathrm{life}}} = \mathrm{N}_{{\mathrm{b}}}/\mathrm{f}_{{\mathrm{H}}} = \mathrm{N}_{{\mathrm{b}}}/{\mathrm{af}}_{{{\mathrm{resp}}}} = \mathrm{N}_{\mathrm{r}}/\mathrm{f}_{{{\mathrm{resp}}}}$. This total number of ‘respiration cycles’, ${\mathrm{N}}_{\mathrm{r}} = \mathrm{f}_{{\mathrm{resp}}} \mathrm{t}_{{{\mathrm{life}}}}$, will be assumed from now on to be the same number for all living organisms; in Conclusions and outlook, we will explore the implications of this conjecture for the origin of aging.

Under the condition of a constant total number ${\mathrm{N}}_{\mathrm{r}}$ of respiration cycles in a lifetime $\mathrm{t}_{{\mathrm{life}}}$, multiplying the new metabolic rate relation^13^ by $\mathrm{t}_{{\mathrm{life}}} /\mathrm{a} = \mathrm{N}_{\mathrm{r}}/{\mathrm{af}}_{{\mathrm{resp}}} = \mathrm{N}_{\mathrm{r}}/{\mathrm{f}}_{{\mathrm{H}}}$ is now equivalent to B${\mathrm{t}}_{{\mathrm{life}}}/{\mathrm{a}} = \epsilon ({\mathrm{T}}) \, \eta _{{\mathrm{O}}_2} \mathrm{N}_{\mathrm{r}} \, \mathrm{M}$. The factor $\epsilon ({\mathrm{T}}) \, \eta _{{\mathrm{O}}_2} = \epsilon _0 \, \eta _{{\mathrm{O}}_2} \, {{\mathrm{e}}^{\small -\mathrm{E}_{\mathrm{a}}/{\mathrm{k}} {\mathrm{T}}}}$ can be rewritten as ${{\mathrm{E}}}_{2019} \, {{\mathrm{e}}^{\small (\frac{1}{\mathrm{T}_{{\mathrm{a}}}}-\frac{1}{{\mathrm{T}}})\frac{{\mathrm{E}}_{\mathrm{a}}}{{\mathrm{k}} } }}$, where ${\mathrm{T}}_{{\mathrm{a}}}$ is a normalizing ‘ambient’ temperature and ${\mathrm{E}}_{2019}= 10^{-4.313} \,{{\mathrm{mlO}}}_2{\mathrm{g}}^{-1} \approx 10^{-3} \, {{\mathrm{Jg}}}^{-1}$ (with the conversion 1 ltr ${\mathrm{O}}_2=20.1 \,\hbox {kJ}$;^17^) is a constant that comes from the best fitted value for the corrected metabolic relation^13^. Therefore, for the metabolic rate relation given in^13^, under the condition of a fixed number of respiration cycles in a lifetime, the following relation is predicted to be valid:

1 $$\begin{aligned} {{\mathrm{exp}}}\Big ({\small \frac{\mathrm{E}_{{\mathrm{a}}}}{\mathrm{k} {\mathrm{T}}}}\Big ) \, \frac{ {\mathrm{B} \, \mathrm{t}_{{\mathrm{life}}}} }{\mathrm{a}} = \mathrm{E}_{2019} \, {{\mathrm{exp}}}\Big ({\small \frac{\mathrm{E}_{\mathrm{a}}}{\mathrm{k} \mathrm{T}_{\mathrm{a}}}}\Big ) \, \mathrm{N}_{\mathrm{r}} \, \mathrm{M} \, . \end{aligned}$$

Equation (1) is a prediction from the mathematically corrected metabolic relation^13^ under the assumption of a constant number ${\mathrm{N}}_{\mathrm{r}}$ of respiration cycles in a lifetime, $\mathrm{t}_{{\mathrm{life}}} = \mathrm{N}_{\mathrm{r}}/\mathrm{f}_{{\mathrm{resp}}}$, which is conjectured in principle for all living organisms. It is important to emphasize that this relation for lifespan energy consumption is not assumed or hypothesized to be fixed, as in the ‘rate of living’ theory; instead, it is derived directly from the metabolic relation under the conjectured invariant ${\mathrm{N}}_{\mathrm{r}}$.In contrast to allometric relations, which are characterized mathematically as having as many dimensional constants (${\mathrm{B}}_0, {\mathrm{M}}_0$, in Kleiber’s case) as there are variables (${\mathrm{B}}, {{\mathrm{M}}}$), the relation given by Eq. (1) has (in principle) 7 variables $({\mathrm{t}}_{{\mathrm{life}}}, \mathrm{B}, \mathrm{a}, \mathrm{M}, \mathrm{E}_{\mathrm{a}}, \mathrm{T}_{\mathrm{a}} \, \& \, {\mathrm{T}})$, one dimensionless number (${\mathrm{N}}_{\mathrm{r}}$) and only two constants with units: one universal constant (the Boltzmann constant k) and one derived by best fitting the energetics of respiration $({\mathrm{E}}_{2019})$; the possible universality of the latter will be discussed in "Conclusions and outlook".

## Empirical support for the corrected relation of the total energy consumed in a lifespan

In this section, we will test the validity and accuracy of the derived relation for total energy consumed in a lifespan [Eq. (1)], which is predicted from the new formulation of the metabolic rate relation^13^ and the conjectured constant total number ${\mathrm{N}}_{\mathrm{r}}$ of respiration cycles per lifespan for all living organisms. For this purpose, we use data from 277 species representing different classes of living organisms, except plants, ranging from unicellular organisms and other ectothermic species to mammals and birds. The data sample, which was compiled and curated by^6^, is also defined and listed in the *Supplementary Material*, including the body mass M, body temperature T and total metabolic energy per lifespan ${\mathrm{B}} \, {\mathrm{t}}_{{\mathrm{life}}}$ (computed from estimations of the basal metabolic rate B and maximum lifespan ${\mathrm{t}}_{{\mathrm{life}}}$ compiled in^6^).

**Figure 1:**
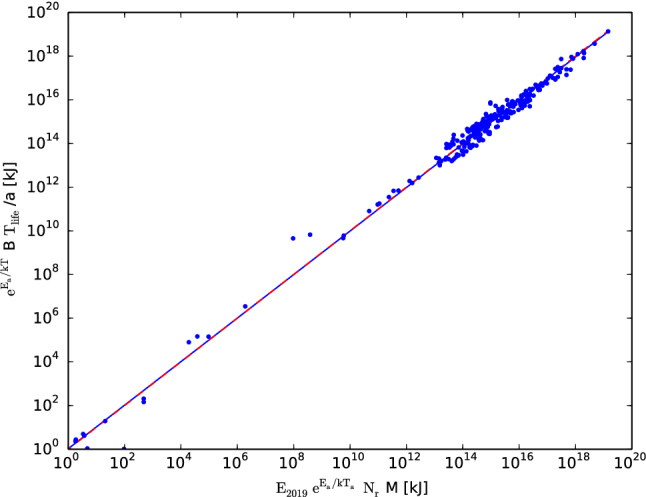
The figure shows the relation predicted by Eq. (1) for 277 living organisms, listed in^6^. The dashed red curve displays the identity given by Eq. (1). The solid blue curve displays the best fitted value of a slope of 0.997 and a normalization of 1.05 for ambient temperature ${\mathrm{T}}_{\mathrm{a}} = 30^{\circ }\hbox {C}$. The two curves are almost indistinguishable in the dynamic range of 20 orders of magnitude.

Figure 1 shows the relation predicted by Eq. (1) for the 277 living organisms listed in^6^, where the activation energy ${\mathrm{E}}_{{\mathrm{a}}}$ was chosen as the average value of 0.63 eV and was determined independently to temperature-normalize the metabolic rates of unicells and poikilotherms to that of endotherms^18,19^. The parameter a was set to the empirically determined values of 4.5 for mammals and 9 for birds^17^, and it was estimated to be 3 for ectotherms with hearts (from the relative size of the hearts in invertebrates; *17*) and was assumed to be unity for ectotherms without hearts ($\sim 20$ species in the dataset), such as unicellular organisms; only a respiration rate can be defined for such organisms, which is equal to the inverse of the characteristic time for the cycle of cellular respiration. The total number ${\mathrm{N}}_{\mathrm{r}} =\mathrm{N}_{{\mathrm{b}}} /\mathrm{a} = 1.62 \times 10^8$ of respiration cycles in a lifetime was determined from the best fitted values for mammals: ${\mathrm{N}}_{{\mathrm{b}}}=7.3 \times 10^8$ heartbeats in a lifetime^16^ and $\hbox {a}=4.5$^17^.

The data displayed in Fig. 1 strongly support the unique relation predicted by Eq. (1), in a dynamic range of 20 orders of magnitude, for different classes of living organisms, from ${\textit{Bacteria}}$ to ${\textit{Elephants Maximum}}$. The solid blue curve displays the best fitted value of a slope of 0.997 and normalization of 1.05, which was determined through ordinary least-squares regression and is almost indistinguishable from the identity predicted by Eq. (1) (dashed red curve). The only free parameter (not predetermined by an independent measurement) in Eq. (1) is the ‘ambient’ temperature ${\mathrm{T}}_{\mathrm{a}} = 30^{\circ }\hbox {C}$, which was chosen only to match the normalization in the best fitted relation (solid blue line) to the identity (dashed red line). Moreover, a slope close to unity (0.997) is independent of the choice of ${\mathrm{T}}_{\mathrm{a}}$, and for example, if we instead decide to preset ${\mathrm{T}}_{\mathrm{a}}$ to the value of mammals ($37^{\circ }\hbox {C}$), this will only change the best fitted normalization value to 1.81. Therefore, the relation between the 5 physiological variables (${\mathrm{t}}_{{\mathrm{life}}}$, B, a, M & T) given by Eq. (1) is confirmed without the choice of any free parameter.

**Figure 2:**
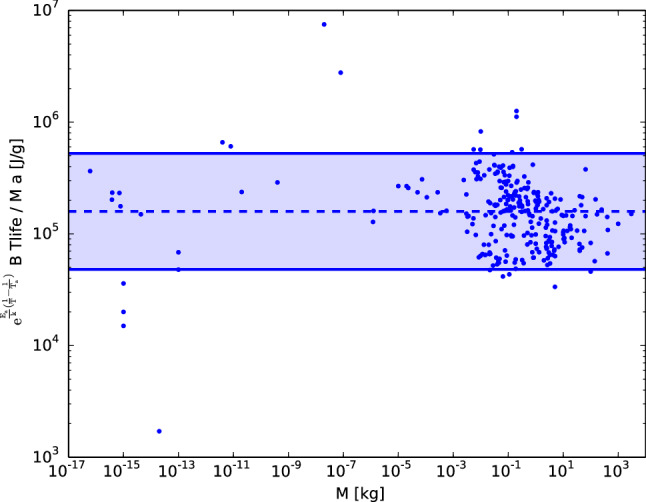
Residuals from the relation predicted by Eq. (1) as a function of the organism’s body mass for 277 living organisms, listed in^6^. The relation has an average scatter of only 0.339 dex around the predicted value (${\mathrm{E}}_{2019} \, \mathrm{N}_{\mathrm{r}}$) denoted by the dashed line. The colored region between the solid curves denotes residuals with less than a geometrical factor of $\pi$ from the relation, and 95% of the points ($2-\sigma$) fulfill this criterion.

Figure 2 shows the residuals from the relation predicted by Eq. (1) as a function of the organism’s body mass. The relation has an average scatter of only 0.339 dex (≈ a factor of 2) around the predicted value (${\mathrm{E}}_{2019} \, {\mathrm{N}}_{\mathrm{r}} = {\mathrm{E}}_{2019} \, \mathrm{N}_{\mathrm{b}}$/a; dashed line in Fig. 2), where 1 dex on a logarithmic - scale refers to an order of magnitude. A fraction of an order of magnitude of scatter is impressively small considering the 20-orders-of-magnitude variations in mass and that the values of ${\mathrm{E}}_{2019} = 10^{-3} \, {{\mathrm{Jg}}}^{-1}$^13^, $\mathrm{N}_{\mathrm{b}}=7.3 \times 10^8$ heartbeats in a lifetime^16^ and $\hbox {a}=4.5$^17^ come from three completely independent measurements in mammals. Moreover, approximately 95% of the points ($2\,\sigma$) have departures from the relation that are less than a factor of $\pi$ ($\approx \, \pm 0.5 \,\hbox {dex}$; colored region between the solid curves in Fig. 2).

The extremely accurate relation displayed in Fig. 2, with departures from the relation of less than a factor of $\pi$, suggests that the only missing parameters are those missing due to geometrical factors (as expected from variations in the geometries among species), which are generally on the order of the dimensionless factor $\pi$. If this is indeed the case, this is probably one of the few relations in life sciences that includes all the relevant controlling parameters and reaches an accuracy comparable to those in the physical sciences. Moreover, Fig. 2 implies there is no clear trend (larger than an e-fold of $\approx \, 0.5 \,\hbox {dex}$) in the interclass comparison from bacteria to the largest mammal.

Interclass variations have been considered the most persuasive evidence against the ‘rate of living’ theory, for example, the interclass comparison of birds with mammals, in addition to some differences within classes, such as those in bats compared with other mammals^2,5^. These exceptions are eliminated in Fig. 2 due to the predicted secondary parameters that are not included in the original formulation (mainly the parameter ${\mathrm{a}}= {\mathrm{f}}_{{\mathrm{H}}}/ \mathrm{f}_{{\mathrm{resp}}}$ for the particular case of comparing birds and bats to mammals). Moreover, Eq. (1) implies a dependence of ${\mathrm{t}}_{{\mathrm{life}}} \propto \frac{\mathrm{M}}{\mathrm{B}} {\mathrm{exp}}\Big ({\small \frac{\mathrm{E}_{\mathrm{a}}}{\mathrm{k} \mathrm{T}_{\mathrm{a}}}}\Big )$ that is in agreement with the three regimens experimentally known to extend the lifespan^20^: a lowered ambient temperature ${\mathrm{T}}_{\mathrm{a}}$ in poikilotherms, a decrease in physical activity in poikilotherms (lower ${\mathrm{f}}_{\mathrm{H}}$ and thus B) and caloric restriction (lower B). Matching these trends with Eq. (1) is particularly important since to explain the varying effects of caloric restriction on lifespan^20^, new theories that differ from the standard ‘rate of living’ theory have been invoked. A particularly relevant idea is the ‘metabolic stability–longevity hypothesis’^37^, where the most important factor involved in the duration of life is not metabolic rate or oxidative stress, but instead metabolic stability. Since Eq. (1) is derived empirically from the combination of two empirical relations and it has excellent agreement with both the data displayed in Fig. 1 and with the regimens known experimentally to extend the lifespan^20^, its predictions can be excellent tests to discriminate between the ‘rate of living’ and the ‘metabolic stability-longevity’ hypotheses.

**Figure 3:**
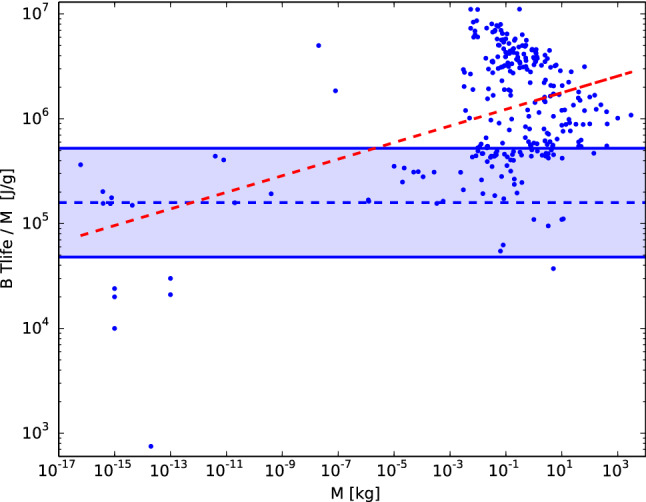
Total metabolic energy exhausted per lifespan per body mass, ${\mathrm{B}} {\mathrm{t}}_{{\mathrm{life}}} / {\mathrm{M}}$, as a function of the organism’s body mass for 277 living organisms, listed in^6^. The dashed red line displays ${\mathrm{B}} {\mathrm{t}}_{{\mathrm{life}}} / \mathrm{M} \propto \mathrm{M}^{0.1}$, the dashed blue line displays the constant value predicted by Eq. (1) ($= {\mathrm{E}}_{2019} \, \mathrm{N}_{\mathrm{r}}$) and the colored region between the solid blue curves denotes the $2-\sigma$ region in Fig. 2 for the purpose of comparing the scatter plot for the standard ‘rate of living’ theory relation with the one predicted by Eq. (1).

The accurate result found in Fig. 2 not only emphasizes the role of secondary parameters but might also seem contradictory to other studies that do not include them. For example^25^, recently found in a larger dataset, with $\sim 8000$ organisms studied, that the total metabolic energy exhausted per lifespan per body mass increases allometrically with body mass. For comparison purposes, in Fig. 3, we plot the total metabolic energy exhausted per lifespan per body mass, ${\mathrm{B}} {\mathrm{t}}_{{\mathrm{life}}} / {\mathrm{M}}$, for the same dataset studied in Fig. 2 but without correcting factors (a, T & ${\mathrm{T}}_{\mathrm{a}}$), showing that in our dataset, this value also increases allometrically with M. This trend again highlights the role of secondary parameters, which is predicted in our formulation.

The dashed red line in Fig. 3 displays the best fitted curve, and for comparison purposes, we also display the value predicted (by Eq. (1); ${\mathrm{E}}_{2019} \, {\mathrm{N}}_{\mathrm{r}}$) and the $2-\sigma$ region of Fig. 2, denoted by the dashed blue line and the colored blue region, respectively. The slope of the dashed red line is slightly weaker ($\sim 0.1$) than the best fitted value ($\sim 0.2$) found in^25^, which can be understood by noting that the dataset in that work is not only larger but also noisier (‘data are of varying quality, with unequal representation across the size spectrum’, as noted by *25*). In particular, in^25^, lifespans are estimated from both the intrinsic physiological potential (i.e., maximum lifespan in captivity) and the extrinsic ecological reality (i.e., average field lifespan), where the second estimation can be considered ‘noisier’ for the purposes of testing the ‘rate of living’ theory. Therefore, the result found in Fig. 2, independence from body mass, is mainly due to the secondary parameters predicted in Eq. (1) and is not a result of the dataset chosen.

## Conclusions and outlook

We showed that the new metabolic rate relation^13^ can be directly linked to the total energy consumed in a lifespan if a constant number ${\mathrm{N}}_{\mathrm{r}}$ of respiration cycles per lifespan is conjectured, and a corrected relation for the total energy consumed in a lifespan was found [Eq. (1)] that can explain the origin of variations in the ‘rate of living’ theory^2,5^ and unify them into a single formulation. It is important to note that Eq. (1) is a direct consequence of combining the two empirical relations mentioned (the new metabolic rate relation and the relation of the total number ${\mathrm{N}}_{\mathrm{r}}$ of respiration cycles per lifespan) and is not an assumption (based on the lifespan energy expenditure per gram) as in the traditional ‘rate of living’ theory^2,5^. We test the validity and accuracy of the predicted relation [Eq. (1)] for the total energy consumed in a lifespan with $\sim 300$ species representing different classes of living organisms, and we find that the relation has an average scatter of only 0.3 dex, with 95% of the organisms having departures of less than a factor of $\pi$ from the relation, despite the difference of $\sim 20$ orders of magnitude in body mass.

Successful testing of predictions is crucial in any proposed theory according to Popper’s deductive method of falsification (27), which is the criterion for identifying a successful scientific theory. Therefore, the success of the predicted Eq. (1) that is displayed in Fig. 1 implies that the corrected metabolic rate relation^13^ has passed an initial test. This prediction also reduces any possible interclass variation in the relation, which has been considered the most persuasive evidence against the ‘rate of living’ theory, to only a geometrical factor and strongly supports the conjectured invariant number ${\mathrm{N}}_{\mathrm{r}} \sim 10^8$ of respiration cycles per lifespan in all living organisms.

Invariant quantities in physics traditionally reflect fundamental underlying constraints, a principle that has also been applied recently to life sciences such as ecology^21,22^. Figure 2 indicates the fact that, for a given temperature, the total lifespan energy consumption per gram per ‘generalized beat’ (${\mathrm{N}}_{{\mathrm{b}}}^{\mathrm{G}} \equiv \mathrm{a} {\mathrm{N}}_{{\mathrm{r}}} = {\mathrm{a}} \,1.62 \times 10^8$) is remarkably constant (around ${\mathrm{E}}_{2019}$), a result that is also in agreement with previous expectations based on (lifespan) basal oxygen consumption at the molecular level^38^. This supports the idea that the overall energetics during the lifespan are the same for all the organisms studied, as it is predetermined by the basic energetics of respiration, and therefore, Rubner’s original picture is shown to be valid without systematic exceptions but in a more general form. Moreover, since the value determined from Fig. 2 is remarkably similar to ${\mathrm{E}}_{2019} {\mathrm{N}}_{\mathrm{r}}$, it can be considered an independent determination of ${\mathrm{E}}_{2019}$, suggesting that ${\mathrm{E}}_{2019}$ is a candidate for being a universal constant and not just a fitting parameter from the corrected metabolic relation^13^.

In addition, we showed here that the invariant total lifespan energy consumption per gram per ‘generalized beat’ comes directly from the existence of another invariant, the approximately constant total number ${\mathrm{N}}_{\mathrm{r}} \sim 10^8$ of respiration cycles per lifetime, effectively converting the ‘generalized beat’ into the characteristic clock during the lifespan. Therefore, the exact physical relation between (oxidative) free radical damage and the origin of aging is most likely related to the striking existence of a constant total number of respiration cycles ${\mathrm{N}}_{{\mathrm{r}}}$ over the lifetime of all organisms, which predetermines the extension of life. Moreover, the relation ${\mathrm{t}}_{{\mathrm{life}}} = \mathrm{N}_{\mathrm{r}}/\mathrm{f}_{{{\mathrm{resp}}}}$ quantifies the ideas of oxidative damage by the respiratory metabolism, which are motivated mainly by biomedical considerations, into a simple mathematical form that could be included in a broader life-history framework; this is needed to produce testable predictions for the ‘free-radical’ hypothesis in the life-history context^28^. Future theoretical and experimental studies that investigate the exact link between the constant number ${\mathrm{N}}_{\mathrm{r}} \sim 10^8$ of respiration cycles per lifespan and the production rates of free radicals (or alternatively, other byproducts of metabolism) should shed light on the origin of aging and the physical cause of natural mortality.

Although this relation ${\mathrm{t}}_{{\mathrm{life}}} = \mathrm{N}_{\mathrm{r}}/\mathrm{f}_{{\mathrm{resp}}}$ has only been empirically examined for mammalian vertebrates, in terms of heartbeats per lifetime, there is evidence to believe that the relative constancy of the number of respiration cycles per lifetime is more widely distributed in the animal kingdom. For example, a reptile such as the Galapagos tortoise with a life expectancy of 177 years and a respiration rate of 3 breaths/min has $2.8 \times 10^8$ breaths per lifetime^29^, which is within a factor of 2 of the value determined for mammals. A more different case is that of birds, which have more heartbeats/lifetime by a factor of 3^30^; this difference is reduced to a factor of 1.5 in terms of breaths/lifetime ($\mathrm{N}_{\mathrm{r}} = \mathrm{N}_{\mathrm{b}}/{\mathrm{a}}$, with $\hbox {a}=9$ for birds and 4.5 for mammals; *17*). Among fish, the average number of heartbeats/lifetime tends to be an order of magnitude less than that in mammals ($\mathrm{N}_{\mathrm{b}} = 7.3 \times 10^8$;^16^), for example, $\mathrm{N}_{\mathrm{b}} = 6.7 \times 10^7$ for trout^31^, but in such cases, the parameter a can be as low as 0.5 (i.e., a heart frequency lower than the respiratory frequency; *32*), again implying a similar ${\mathrm{N}}_{\mathrm{r}} \,(= \mathrm{N}_{\mathrm{b}}/{\mathrm{a}} = 1.3 \times 10^8)$. A more extreme difference in terms of heartbeats is the tiny Daphnia, which uses up to $1.7 \times 10^7$ heartbeats (at 25 C) in a short lifespan of 30 days^33^. Simple invertebrates, such as Daphnia, do not have a complex respiratory system with lungs and obtain oxygen for respiration through diffusion, but a “breath frequency” can be estimated from its respiration rate ($\sim \mu {\mathrm{l}} {\mathrm{O}}_2 \hbox {hr}^{-1}$;^34^) divided by ${\mathrm{E}}_{2019} M$ (with ${\mathrm{M}} \sim 100 \mu {\mathrm{g}}$;^35^), giving ${\mathrm{N}}_{\mathrm{r}} = 1.5 \times 10^8$ respiration cycles per lifetime. In summary, a difference of two orders of magnitude in total heartbeats (between Daphnia and birds) is reduced to less than a factor of 2 in breaths per lifetime, further supporting that all organisms seem to live for the same span in units of respiration cycles (${\mathrm{N}}_{\mathrm{r}} \sim 10^8$).

It has also been suggested that an analogous invariant originates at the molecular level^23^, the number of ATP turnovers of the molecular respiratory complexes per cell in a lifetime, which, from an energy conservation model that extends metabolism to intracellular levels, is estimated to be $\sim 1.5 \times 10^{16}$^23^. A similar number can be determined by taking into account that human cells require the synthase of approximately 100 moles of ATP daily, equivalent to $7 \times 10^{20}$ molecules per second. For $\sim 3 \times 10^{13}$ cells in the human body and for a respiration rate of 15 breaths per minute, this gives $\sim 9 \times 10^{7}$ ATP molecules synthesized per cell per breath, which for the invariant total number ${\mathrm{N}}_{\mathrm{r}}$ of respiration cycles per lifetime found in this work, rises to the same number of $\sim 1.5 \times 10^{16}$ ATP turnovers in a lifetime per cell, showing the equivalence between both invariants and linking ${\mathrm{N}}_{\mathrm{r}}$ to the energetics of respiratory complexes at the cellular level.

The excellent agreement between the predicted relation [Eq. (1)] and the data across all types of organisms emphasizes the fact that lifespan indeed depends on multiple factors (B, a, M, T & $\mathrm{T}_{\mathrm{a}}$) and strongly supports the methodology presented in this work of multifactorial testing, as shown in Fig. 1, since quantities in life sciences generally suffer from a confounding variable problem. An example of this problem, illustrated by individually testing each of the relevant factors, is given in^24^, which for a large (and noisy) sample test for ${\mathrm{t}}_{{{\mathrm{life}}}} \propto 1/B$ shows no clear correlation. From Eq. (1), it is clear that in an uncontrolled experiment, the dependence on the rest of the parameters (M, a, T, & ${\mathrm{T}}_{\mathrm{a}}$) might eliminate the dependence on the metabolic rate B (in fact, this may be for the same reason that Rubner’s work^7^ focused on the mass-specific metabolic rate B/M instead of B). This work^24^ finds only a residual inverse dependence of ${\mathrm{t}}_{{\mathrm{life}}}$ on the ambient temperature ${\mathrm{T}}_{{\mathrm{a}}}$ for ectotherms, which is expected according to Eq. (1) $\Big (\mathrm{t}_{{\mathrm{life}}} \propto {\mathrm{exp}}\Big ({\small \frac{\mathrm{E}_{\mathrm{a}}}{\mathrm{k} {\mathrm{T}}_{\mathrm{a}}}}\Big ) \Big )$.

Finally, the empirical support in favor of Eq. (1) allows us to perform several estimations regarding how much the energy consumption will vary with changing physical conditions on Earth. For example, computing by how much the energy consumption will vary in biomass performing aerobic respiration as the Earth’s temperature increases is relevant in the current context of possible global warming. This is given by the factor ${\mathrm{exp}}\Big [{\small \frac{\mathrm{E}_{\mathrm{a}}}{{\mathrm{k}}} \Big (\frac{1}{ {\mathrm{T}}}} - {\small \frac{1}{ {\mathrm{T}}+1}}\Big ) \Big ]$, which for an activation energy of ${\mathrm{E}}_{\mathrm{a}} = 0.63 \,\hbox {eV}$ and a temperature of $30^{\circ }\hbox {C}$ implies an increase of 8.3% in energy consumption per 1 degree increase in the average Earth temperature. This result can be straightforwardly applied in ectotherms since their body temperatures adapt to the environmental temperature (${\mathrm{T}}={\mathrm{T}}_{\mathrm{a}}$), but its implications for endothermic organisms are less clear. Another relevant estimation is to compute by how much B${\mathrm{t}}_{{\mathrm{life}}}$/M would vary from Eq. (1) (i.e., the difference between Figs. 2 and 3) as a function of body temperature (T) and the ratio of heart rate to respiratory rate ($\mathrm{a}= \mathrm{f}_{\mathrm{H}}/ {\mathrm{f}}_{{\mathrm{resp}}}$). Variations in B${\mathrm{t}}_{{\mathrm{life}}}$/M are relevant since this is a key quantity in the estimation of the energy allocation to fitness, which aims to explain in terms of trade offs the so-called ‘Equal Fitness Paradigm’^39^ that concerns why most organisms in the biosphere are more or less equally fit, other than the diversity seen in the size, form and function of living organisms on Earth.

In the near future, our plan is to generate a (metabolic) theory starting from the new metabolic rate relation^13^ by assuming that it is the controlling rate in ecology in order to explain a variety of ecological phenomena in a similar fashion as the metabolic theory of ecology^18^ does using Kleiber’s law. A first step in this direction looks very promising^40^, as it can show that ontogenetic growth can be described by a universal growth curve without the aid of fitting parameters, can explain the origin of several ‘Life History Invariants’^21^ and can show how the heart rate may actually set several biological times (i.e., lifespan and generation time) and even some ecological rates (i.e., The Malthusian parameter).

## Supplementary Information

Supplementary Information 1.
